# Enhanced anti-tumor efficacy of 5-aminolevulinic acid-gold nanoparticles-mediated photodynamic therapy in cutaneous squamous cell carcinoma cells

**DOI:** 10.1590/1414-431X20208457

**Published:** 2020-04-27

**Authors:** Yu-fei Chi, Jing-jing Qin, Zhi Li, Qin Ge, Wei-hui Zeng

**Affiliations:** 1 Department of Dermatology, The Second Affiliated Hospital of Xi'an Jiaotong University, Xi'an, China; 2 Department of Dermatology, Xijing Hospital, Fourth Military Medical University, Xi’an, China; 3 Department of Dermatology, Forest Industry Worker Hospital of Shaanxi Province, Xi'an, China; 4 Department of Dermatology, Qingdao Municipal Hospital, Qingdao, China; 5 Department of Dermatology, Jingmen No.1 People's Hospital, Jingmen, China

**Keywords:** Photodynamic therapy, 5-ALA-GNPs, Cell viability, Cell apoptosis, Cell invasion and migration, Cutaneous squamous cell carcinoma

## Abstract

The objective of this study was to investigate whether the conjugation of gold nanoparticles (GNPs) to 5-aminolevulinic acid (5-ALA) could enhance the anti-tumor efficiency of photodynamic therapy (PDT) in epidermoid carcinoma cells. The mRNA and protein expression levels were determined by quantitative real-time PCR and western blot, respectively. Cell viability, apoptosis, invasion, and migration were determined by MTT assay, flow cytometry, transwell invasion assay, and migration assay, respectively. Singlet oxygen generation was detected by the singlet oxygen sensor green reagent assay. Our results showed that PDT with 5-ALA and GNPs-conjugated 5-ALA (5-ALA-GNPs) significantly suppressed cell viability, increased cell apoptosis and singlet oxygen generation in both HaCat and A431 cells, and PDT with 5-ALA and 5-ALA-GNPs had more profound effects in A431 cells than that in HaCat cells. More importantly, 5-ALA-GNPs treatment potentiated the effects of PDT on cell viability, cell apoptosis, and singlet oxygen generation in A431 cells compared to 5-ALA treatment. Further *in vitro* assays showed that PDT with 5-ALA-GNPs significantly decreased expression of STAT3 and Bcl-2 and increased expression of Bax in A431 cells compared with PDT with 5-ALA. In addition, 5-ALA-GNPs treatment enhanced the inhibitory effects of PDT on cell invasion and migration and Wnt/β-catenin signaling activities in A431 cells compared to 5-ALA treatment. In conclusion, our results suggested that GNPs conjugated to 5-ALA significantly enhanced the anti-tumor efficacy of PDT in A431 cells, which may represent a better strategy to improve the outcomes of patients with cutaneous squamous cell carcinoma.

## Introduction

Cutaneous squamous cell carcinoma (cSCC) is the second most frequently diagnosed non-melanoma skin cancer worldwide (after basal cell carcinoma) (1) and the incidence of cSCC is increasing. Surgical excision remains the gold standard treatment for localized cSCC, which can be managed through excisional surgery, cryosurgery, curettage and desiccation, Mohs micrographic surgery, or micrographic surgery (2). However, these approaches have a number of side effects, such as scarring, development of sensitive areas and painful lesions, and are not suitable for elderly patients (3). Moreover, treatment of extensive locally destructive or metastatic disease is still a challenge and treatments are seldom curative. As the primary non-surgical option, radiation therapy also plays an important role in the treatment of cSCC, especially tumors on the lip and eyelid (4). However, radiation therapy commonly induces side effects including nausea, vomiting, erythema, epidermal atrophy, telangiectasia, soft-tissue necrosis, and radiation-induced malignancies that some patients find difficult to tolerate (5).

Photodynamic therapy (PDT) is an effective, non-invasive procedure that has been clinically approved for treating cancer and other malignant diseases. PDT offers the advantages of minimal invasiveness, better cosmetic outcomes, low morbidity, minimal functional disturbances, and is well tolerated and can be applied repeatedly at the same site (6). Thus, PDT is widely used in the treatment of superficial skin cancers including actinic keratoses (7), Bowen's disease (8), and superficial basal cell carcinomas (9). In addition, PDT has been applied in the treatment with other types of human malignancies such as cervical cancer (10), breast cancer (11), prostate cancer (12), and glioma (13). With its range of indications continually expanding, PDT has also demonstrated potential as a treatment for cSCC (14).

PDT involves three essential components: visible-to-near-infrared (vis-NIR) light, a photosensitizer (PS), and sufficient amounts of oxygen (15). Diseased tissues can be highly selectively eradicated on activation of the non-toxic PS by irradiation. The second-generation PS 5-aminolevulinic acid (5-ALA) and its derivative methyl aminolevulinate have been approved by the Food and Drug Administration and are commonly used as topical PS (16). Although the use of 5-ALA-PDT for treating skin cancers is growing in popularity, a number of challenges must be overcome to improve the efficacy of PDT. Firstly, 5-ALA is hydrophilic and thus has limited ability to penetrate cell membranes, which restricts the effectiveness of topical 5-ALA PDT. The low selectivity of 5-ALA for malignant cells and low levels of singlet oxygen generated also limit the clinical application of PDT (17). Furthermore, the instability of 5-ALA under physiological conditions reduce its photophysical properties and photodamage activity (18).

Gold nanoparticles (GNPs) possess a number of excellent features for use as drug delivery systems, including their tailorable size, stability, biocompatibility, and surface chemistry. Kim et al. (19) showed that tamoxifen conjugated to GNPs showed enhanced cell uptake by membrane-mediated diffusion and promoted the inhibitory effects of tamoxifen on breast cancer cells. Zhang et al. (20) developed a method for increasing paclitaxel solubility by covalent attachment to GNPs via DNA linkers, and the conjugates were more effective to inhibit viability of breast cancer cells and uterine sarcoma cells. Recently, Kwon et al. (21) demonstrated that Pu-18-N-butylimide-NMGA-GNP conjugated with PDT is effective against hepatocellular carcinoma progression.

In this study, we investigated whether 5-ALA-conjugated GNPs could enhance the anti-cancer efficiency of PDT in cutaneous squamous cell carcinoma as determined by *in vitro* functional assays and explored the underlying molecular mechanisms.

## Material and Methods

### Synthesis of 5-ALA-GNPs

GNPs were synthesized via the branched polyethylenimine (BPEI) method. To obtain positively charged GNPs, BPEI was used to reduce HAuCl_4_ into gold atoms and employed as a stabilizer. Briefly, 0.05 g BPEI and 4 mL HAuCl_4_ (25 mmol/L) were mixed with ultrapure water (total volume, 50 mL) at 80°C, the solution was mixed until the color changed from yellow to dark red, and centrifuged at 25,000 *g* (CP 100 WX, HITACHI, Japan) for 30 min at 4°C to pellet the GNPs. The supernatant was discarded and 10 mL ultrapure water was added to preserve the GNPs.

The 5-ALA solution was prepared by dissolving 0.0336 g 5-ALA in 2 mL ultrapure water to obtain a concentration of 50 mmol/L in the dark. The GNPs and 5-ALA were filtered through 0.22-µm filters. The 5-ALA-GNPs were obtained by mixing 5-ALA and GNPs in a 1:2 ratio for 3 min; HEPES (20 mM) was used as a buffer to adjust the pH to 7.8.

### Characterization of 5-ALA-GNPs

The morphology of GNPs and 5-ALA-GNPs were investigated via high-resolution transmission electron microscopy (TEM; JEM-200CX, Hitachi, Japan). The diameter of the GNPs and the 5-ALA-GNPs were measured using a ZetaSizer Nano ZS90 instrument (Malvern Instruments, UK). The UV-Vis absorption spectra of GNPs and 5-ALA-GNPs were examined using an ultraviolet-visible spectrophotometer (DU-64, Jasco, Japan).

### Culture of epidermoid carcinoma A431 cells and HaCat cells

A431 and HaCat cells were purchased from the Shanghai Cell Library of the Chinese Science Academy (China). A431 cells and HaCat cells were cultured in DMEM (Dulbecco's modified Eagle's medium, USA) containing 10% fetal bovine serum (FBS; Gibco, Thermo Fisher Scientific, USA), 100 U/mL penicillin, and 100 U/mL streptomycin at 37°C in a humidified atmosphere of 5% CO_2_. The culture medium was refreshed every 2 days.

### *In vitro* PDT

A431 cells or HaCat cells were seeded into 96-well plates in triplicate at 1×10^5^ cells per well. Cells were incubated with phosphate-buffered saline (PBS), GNPs, 5-ALA (2, 4, and 8 mM) or 5-ALA-GNPs (2, 4, and 8 mM) for 6 h in the dark, then irradiated at 621 nm using LEDs for 1.5 h. A red LED light source (central wavelength=621 nm; full width at half maximum=15 nm; luminous intensity 4000–5000 mcd; Xi'an Jiatong University, China) containing 96 LEDs with maximal emission to achieve a greater penetration depth and improve the efficacy of PDT was employed, and the energy fluency of the light sources was adjusted to 1 mW/cm^2^ using a variable resistor in series.

### Morphology assessment and cell viability analysis (MTT assay and Alamar blue assay)

At 24 h after irradiation, the morphology of the A431 cells and HaCat cells was observed via inverted microscopy (TE2000-U, Nikon, Japan). The MTT assay was employed to quantify cell viability. Briefly, 24 h after irradiation, the media in the 96-well plates was changed to 100 µL drug-free DMEM medium and 20 µL MTT (5 mg/mL, Sigma, USA) and the cells were incubated in the dark for 4 h. The media was then removed and 50 µL of dimethyl sulfoxide (Sigma) was added to each well. Absorbance values were determined at 570 nm using a microplate reader (Wellscan MK3; Labsystems Dragon, Finland). For the Alamar blue assay, 24 h after irradiation, Alamar blue (10% v/v) was added for an additional 3 h before fluorescence was measured in triplicates for each sample with a fluorescence plate reader with excitation and emission at 560 and 590 nm, respectively (Wellscan MK3).

### Apoptosis assay

At 24 h after irradiation, the cells were harvested and incubated with 5 µL of FITC-conjugated Annexin V and 5 µL of propidium iodide for 15 min (Sigma) according to manufacturer's instructions at room temperature in the dark. The proportions of apoptotic cells were quantified using a FACS Calibur flow cytometer and Cellquest software (BD Biosciences, USA).

### Quantitative analysis of singlet oxygen generation

Singlet oxygen sensor green reagent (SOSGR) was employed as a singlet oxygen-tracking agent to assess intracellular singlet oxygen generation. SOSGR emits green fluorescence at 525 nm in the presence of singlet oxygen. For the SOSGR assay, the cells were incubated with different reagents for 6 h in the dark. Samples were then centrifuged at 12,000 *g* for 5 min at 4^o^C followed by washing with PBS to remove remaining materials, and re-suspended in sodium dodecyl sulfate lysis buffer. The samples were then incubated for 20 min on ice and centrifuged at 12,000 *g* for 5 min at 4^o^C. The suspension solutions of these centrifuged samples were collected and mixed with SOSGR reagent followed by light irradiation. SOSGR fluorescence was quantified at 24 h after irradiation using a fluorescence spectrophotometer (Hitachi F-4500, Japan) with a 525 nm excitation laser and near-infrared (NIR) detector (22).

### Quantitative real-time PCR analysis

Total mRNA was isolated from cells by using TRIzol (Takara, China) and cDNA was synthesized using the SuperScript first-strand synthesis system (Invitrogen, USA). Relevant genes were amplified using the primers listed in Table 1 on an ABI 7500 detector (Applied Biosystems, USA) using the SYRB Green Master Mix kit (Takara). GAPDH was used as the internal reference for the relative expression of mRNA, and the relative expression levels of the genes were calculated by 2^-ΔΔCt^ method.

**Table 1:** Primer sequences for qRT-PCR.

Genes	Primer sequences
STAT3 (forward)	5′-GAGAAGCATTGTGAGTGAGC-3′
STAT3 (reverse)	5′-CGGTCCAGGCAGATGTTG-3′
Bcl-2 (forward)	5′-TTTGAGTTCGGTGGGGTCATG-3′
Bcl-2 (reverse)	5′-TCACTTGTGGCTCAGATAGGC-3′
Bax (forward)	5′-AACTGGTGCTCAAGGCCCTG-3′
Bax (reverse)	5′-GGGTGAGGAGGCTTGAGGAG-3′
β-catenin (forward)	5′-GCTTGGAATGAGACTGCTGA-3′
β-catenin (reverse)	5′-CTGGCCATATCCACCAGAGT-3′
c-myc (forward)	s5′-AGCGACTCTGAGGAGGAACA-3′
c-myc (reverse)	5′-TCCAGCAGAAGGTGATCCA-3′
cyclin D1 (forward)	5′-TGCCACAGATGTGAAGTTCATT-3′
cyclin D1 (reverse)	5′-CAGTCCGGGTCACACTTGAT-3′
GAPDH (forward)	5′-CAAGGTCATCCATGACAACTTTG-3′
GAPDH (reverse)	5′-GTCCACCACCCTGTTGCTGTAG-3′

### Western blot analysis

Proteins from cells were extracted using the RIPA lysis buffer (Bio-Rad, USA) and the concentrations of the extracted proteins were measured using Bradford protein dye reagent (Bio-Rad) according to the manufacturer's protocol. Equal amounts of proteins were resolved by 10% SDS-PAGE and transferred to a PVDF membrane. After blocking with 5% non-fat milk at room temperature for 1 h, the membrane was incubated with antibodies against STAT3, Bcl-2, Bax, β-catenin, c-myc, cyclin D1, and β-actin (Abcam, USA) at 4°C overnight. After that, the membrane was further incubated with respective horseradish peroxidase-conjugated secondary antibodies for 1 h at room temperature. The blots were visualized using an enhanced chemiluminescence kit (Thermo Fisher Scientific, USA) according to the manufacturer's protocol.

### Transwell invasion and migration assays

Cell invasion and migration were evaluated using Transwell chambers (8-µm pore size; Millipore, USA). For the transwell invasion assay, the transwell inserts were pre-coated with Matrigel (Sigma), and for the transwell migration assay, the transwell inserts were without Matrigel. Briefly, cells were incubated with phosphate-buffered saline (PBS), GNPs, 5-ALA (8 mM), or 5-ALA-GNPs (8 mM) for 6 h in the dark, and 24 h after irradiation, the cells in 200 µL serum-free medium were seeded onto the upper transwell chambers, while the lower transwell chambers were filled with 500 µL medium supplemented with 10% FBS as a chemoattractant. After incubation for 24 h at 37°C, the cells on the upper surface of the transwell chambers were removed by cotton wool, and the invaded and migrated cells on the lower surface were fixed with 70% ethanol followed by staining with 0.5% crystal violet. The number of invaded and migrated cells were counted under a light microscope by randomly selecting five fields.

### Statistical analysis

Data were analyzed using GraphPad Prism 6.0 (GraphPad Software, USA). Data are reported as means±SD. Significant differences between treatment groups were analyzed using Student's *t-*test, one-way ANOVA (Bonferroni's *post hoc* test), or two-ANOVA (Bonferroni's *post hoc* test), as appropriate. P values less than 0.05 were considered statistically significant.

## Results

### Characterization of 5-ALA-GNPs

**Figure 1:**
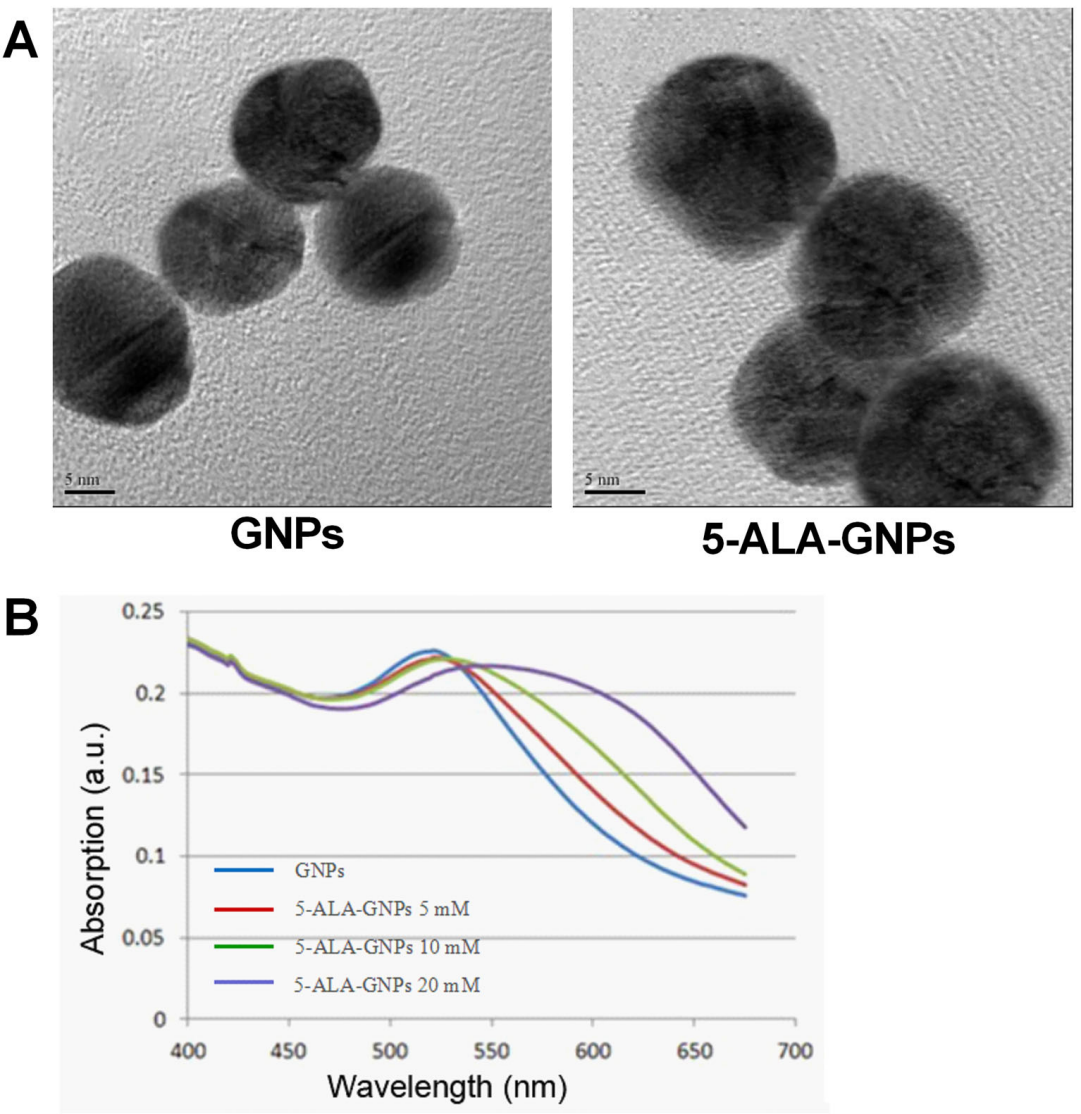
Transmission electron microscopy images and absorption spectra of gold nanoparticles (GNPs) and 5-aminolevulinic acid-(5-ALA)-GNPs. **A**, Transmission electron microscopy images of GNPs and 5-ALA-GNPs. Scale bars, 5 nm. **B**, Absorption spectra of GNPs and 5-ALA-GNPs.

Transmission electron microscopy showed GNPs were uniform spheres with a smooth surface; agglomeration was not observed (Figure 1A, left panel). The 5-ALA-GNPs and GNPs had similar surface morphologies. However, 5-ALA-GNPs had a larger diameter (18.2±1.4 nm) than GNPs (16.1±1.1 nm) (Figure 1A, right panel), indicating 5-ALA was effectively and stably combined with the GNPs.

The UV-Vis absorption spectra of GNPs and various concentrations of 5-ALA-GNPs were recorded (Figure 1B). The absorption peak for 5-ALA-GNPs at a 5-ALA concentration of 5 mmol/L was approximately 530 nm. As the concentration of 5-ALA increased, the absorption peak tended to a red shift: the absorption peaks for 5-ALA-GNPs at 5 and 10 mmol/L 5-ALA were similar, and the absorption peak for 5-ALA-GNPs at 20 mmol/L was about 550 nm. This red shift indicated that the 5-ALA-GNP conjugates were unstable at high concentrations of 5-ALA. Thus, 5-ALA was prepared at concentrations of 2 mmol/L, 4 mmol/L, and 8 mmol/L for the subsequent *in vitro* tests.

### Morphology of HaCat and A431 cells

**Figure 2:**
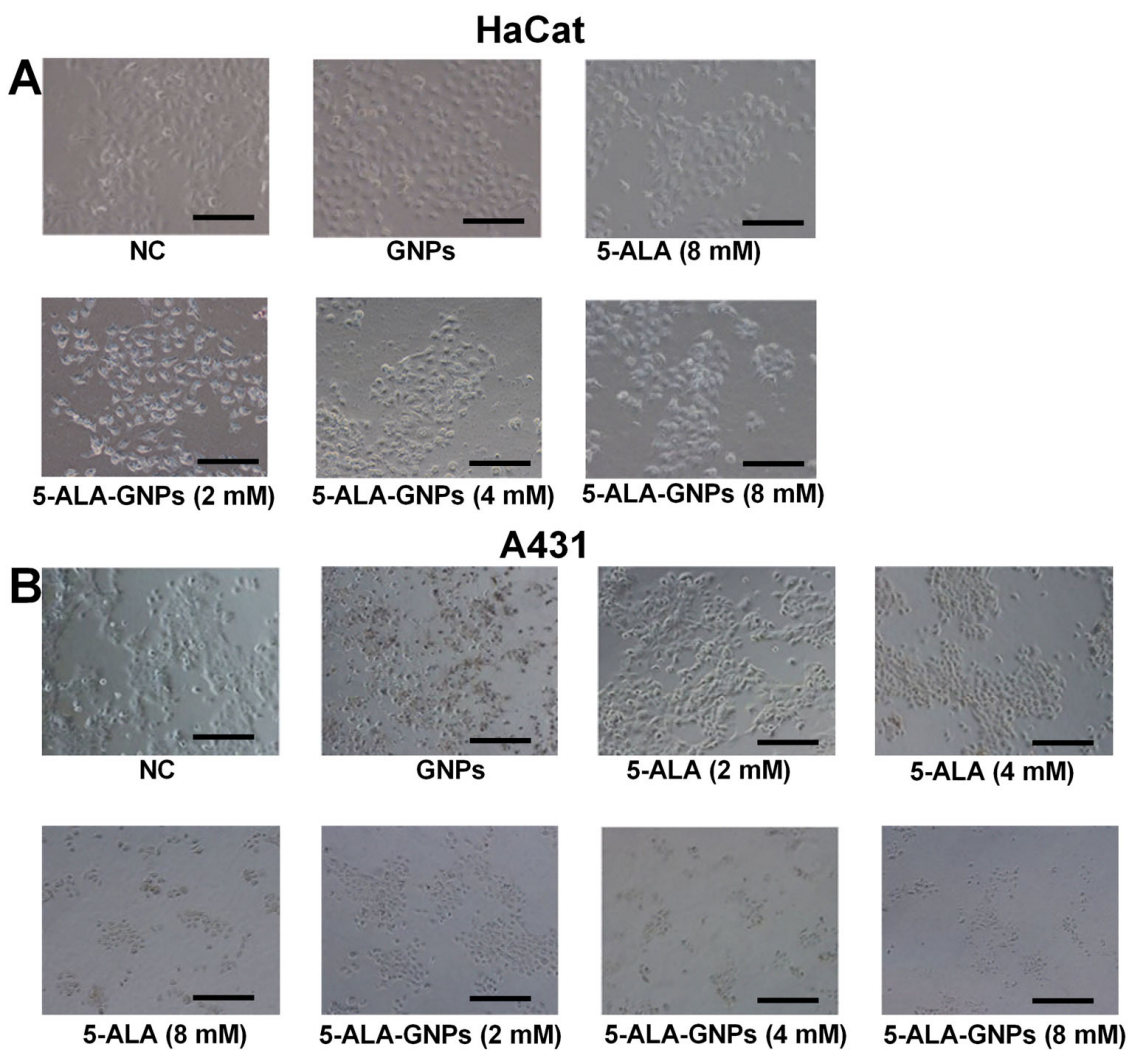
Morphology of HaCat and A431 cells after *in vitro* photodynamic therapy (PDT) with 5-aminolevulinic acid (5-ALA) or 5-ALA-gold nanoparticles (GNPs). **A**, Morphology of HaCat cells after being treated with phosphate-buffered saline (PBS) (negative control (NC)), GNPs, or *in vitro* PDT with 5-ALA (8 mM) or 5-ALA-GNPs (2, 4, and 8 mM). Scale bars, 200 µm. **B**, Morphology of A431 cells after being treated with PBS (NC), GNPs, or *in vitro* PDT with 5-ALA (2, 4, and 8 mM) or 5-ALA-GNPs (2, 4, and 8 mM). Scale bars, 200 µm.

Inverted light microscopy revealed that HaCat cells from the NC group expanded and gradually fused into each other, and the adherent cells were formed relatively homogeneous with a long spindle shape. GNPs treatment alone had no obvious effect on the morphology of HaCat cells compared to that from the NC group. PDT-treated (5-ALA or 5-ALA-GNPs) HaCat cells generally had a fusiform or polygonal shape without obvious pathological changes. However, cell debris and a reduction in cell volume were observed in the 5-ALA-GNPs (8 mM) group (Figure 2A).

The A431 cells from the NC group had a polygonal shape and were distributed evenly with clear boarders, and GNPs treatment had no significant effect on the morphology of A431 cells compared to those from the NC group. Cell shrinkage was observed in the 5-ALA (2, 4, and 8 mM) group. In the 5-ALA-GNPs group, nuclear pyknosis and cell shrinkage were observed, and the cells had an irregular polygonal shape. At high concentrations (4 and 8 mM) of 5-ALA-GNPs, the nuclei manifested the typical characteristics of apoptosis (Figure 2B).

### Effects of *in vitro* PDT on cell viability, cell apoptosis, and singlet oxygen generation in HaCat and A431 cells

**Figure 3:**
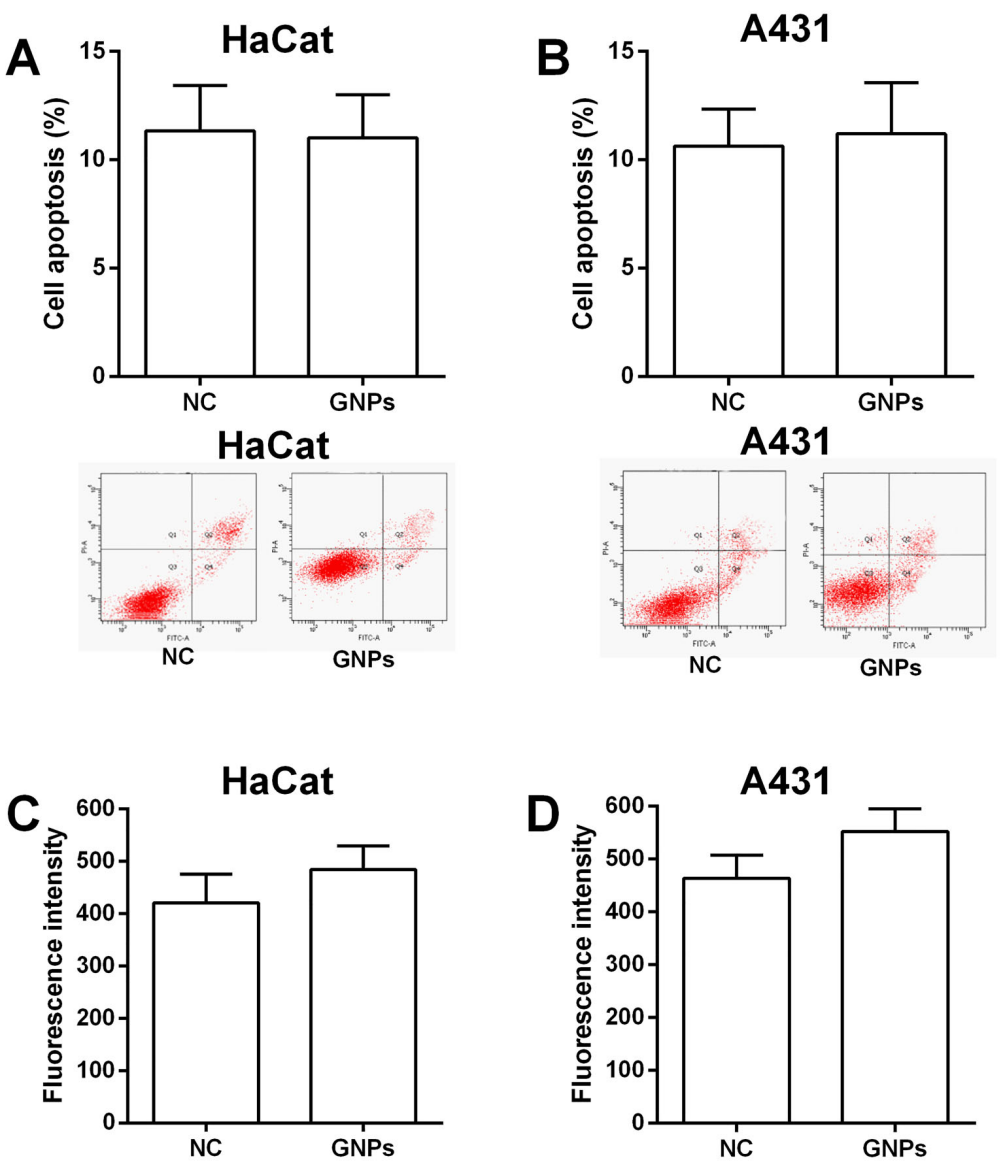
Effects of gold nanoparticles (GNPs) on cell apoptosis and singlet oxygen generation. Cell apoptosis of (**A**) HaCat and (**B**) A431 cells after being treated with phosphate-buffered saline (PBS (NC)) or GNPs was determined by flow cytometry (unpaired *t*-test). Singlet oxygen generation of (**C**) HaCat and (**D**) A431 cells after being treated with PBS (NC) or GNPs was determined by SOSGR assay (unpaired *t*-test). Data are reported as means±SD (n=3).

Treatment with different concentrations of 5-ALA for 6 h in the dark had no significant effect on the cell viability of HaCat and A431 cells (Supplementary Figure S1). In the PDT studies, HaCat and A431 cells were treated with PBS (NC), GNPs, 5-ALA (2, 4, and 8 mM), and 5-ALA-GNPs (2, 4, and 8 mM) for 6 h in the dark, and then irradiated at 628 nm for 1.5 h. At 24 h after irradiation, cells were subjected to different *in vitro* functional assays. The MTT assay showed GNPs treatment had no significant effect on cell proliferative ability of both HaCat and A431 cells (data not shown). Flow cytometry showed no significant differences in cell apoptotic rates between NC and GNPs groups (Figure 3A and B). In addition, GNPs treatment did not affect the singlet oxygen generation of HaCat and A431 cells compared to the NC group (Figure 3C and D).

**Figure 4:**
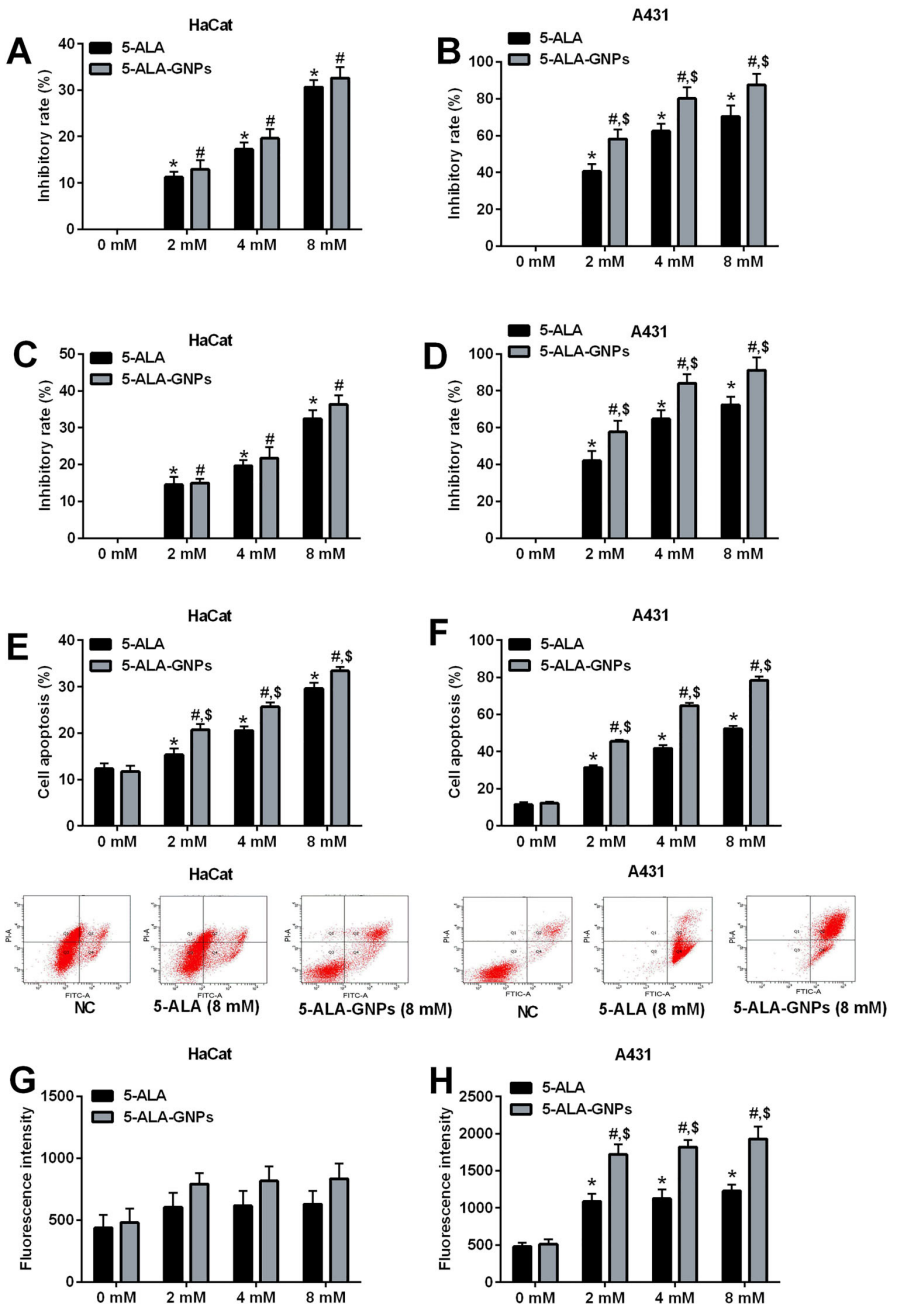
Effects of *in vitro* photodynamic therapy (PDT) on cell viability, cell apoptosis, and singlet oxygen generation in HaCat and A431 cells. Cell viability of (**A**) HaCat and (**B**) A431 cells after *in vitro* PDT with 5-aminolevulinic acid (5-ALA (2, 4, and 8 mM), 5-ALA-gold nanoparticles (GNPs (2, 4, and 8 mM)) or phosphate-buffered saline (PBS (NC)) were determined by MTT assay. Cell viability of (**C**) HaCat and (**D**) A431 cells after similar treatment were determined by Alamar blue assay. Cell apoptosis of (**E**) HaCat and (**F**) A431 cells after similar treatment were determined by flow cytometry. Singlet oxygen generation of (**G**) HaCat and (**H**) A431 cells after similar treatment were determined by SOSGR assay. Data are reported as means±SD. *P<0.05 compared to the 5-ALA (0 mM) group; ^#^P<0.05 compared to 5-ALA-GNPs (0 mM) group; ^$^P<0.05, 5-ALA-GNPs group *vs* 5-ALA group (two-way ANOVA, n=3).

In the HaCat cells, PDT with 5-ALA suppressed cell viability in a concentration-dependent manner, with inhibitory rates being 11.23±1.22% (2 mM 5-ALA), 17.23±1.44% (4 mM 5-ALA), and 30.65±1.51% (8 mM 5-ALA), while 5-ALA-GNPs groups also showed inhibitory rates of 12.93±1.97% (2 mM 5-ALA-GNPs), 19.62±2.01% (4 mM 5-ALA-GNPs), and 32.62±2.33% (8 mM 5-ALA-GNPs). However, no significant differences were found in the inhibitory rates between 5-ALA and 5-ALA-GNPs groups (Figure 4A). In the A431 cells, PDT with 5-ALA and 5-ALA-GNPs suppressed cell viability in a concentration-dependent manner, with inhibitory rates being much higher than that in HaCat cells (Figure 4B). Comparing the 5-ALA groups and the 5-ALA-GNPs groups with the same concentrations, the inhibitory rates in 5-ALA-GNPs groups were significantly higher than those in the 5-ALA groups (Figure 4B). The effects of different treatments on cell viability were further confirmed by the Alamar blue assay (Figure 4C and D). Cell apoptosis after PDT in HaCat and A431 cells was evaluated by flow cytometry. PDT with 5-ALA and 5-ALA-GNPs increased the cell apoptosis in HaCat and A431 cells in a concentration-dependent manner. Cell apoptotic rates of A431 cells after PDT with 5-ALA and 5-ALA GNPs were much higher than those in HaCat cells. Additionally, 5-ALA-GNPs treatment significantly potentiated the effects of PDT on the cell apoptosis of HaCat and A431 cells compared to that with 5-ALA treatments (Figure 4E and F; Supplementary Figure S2).

PDT with 5-ALA and 5-ALA-GNPs had no significant effect on the singlet oxygen generation in HaCat cells (Figure 4G). On the other hand, PDT with 5-ALA increased the singlet oxygen generation in a concentration-dependent manner, with fluorescent intensity being 1086±107 (2 mM 5-ALA), 1125±126 (4 mM 5-ALA), and 1229±82 (8 mM 5-ALA), while PDT with 5-ALA-GNPs also significantly increased oxygen generation with fluorescent intensity being 1720±17 (2 mM 5-ALA-GNPs), 1820±96 (4 mM 5-ALA-GNPs), and 1930±162 (8 mM 5-ALA-GNPs) in a concentration-dependent manner (Figure 4H). When comparing the 5-ALA and 5-ALA-GNPs groups with the same concentrations, the singlet oxygen generation levels in the 5-ALA-GNPs groups were significantly higher than those in the 5-ALA groups (Figure 4H).

### Effects of *in vitro* PDT on expression of STAT3, Bcl-2, Bax, cell invasion, and migration in A431 cells

**Figure 5:**
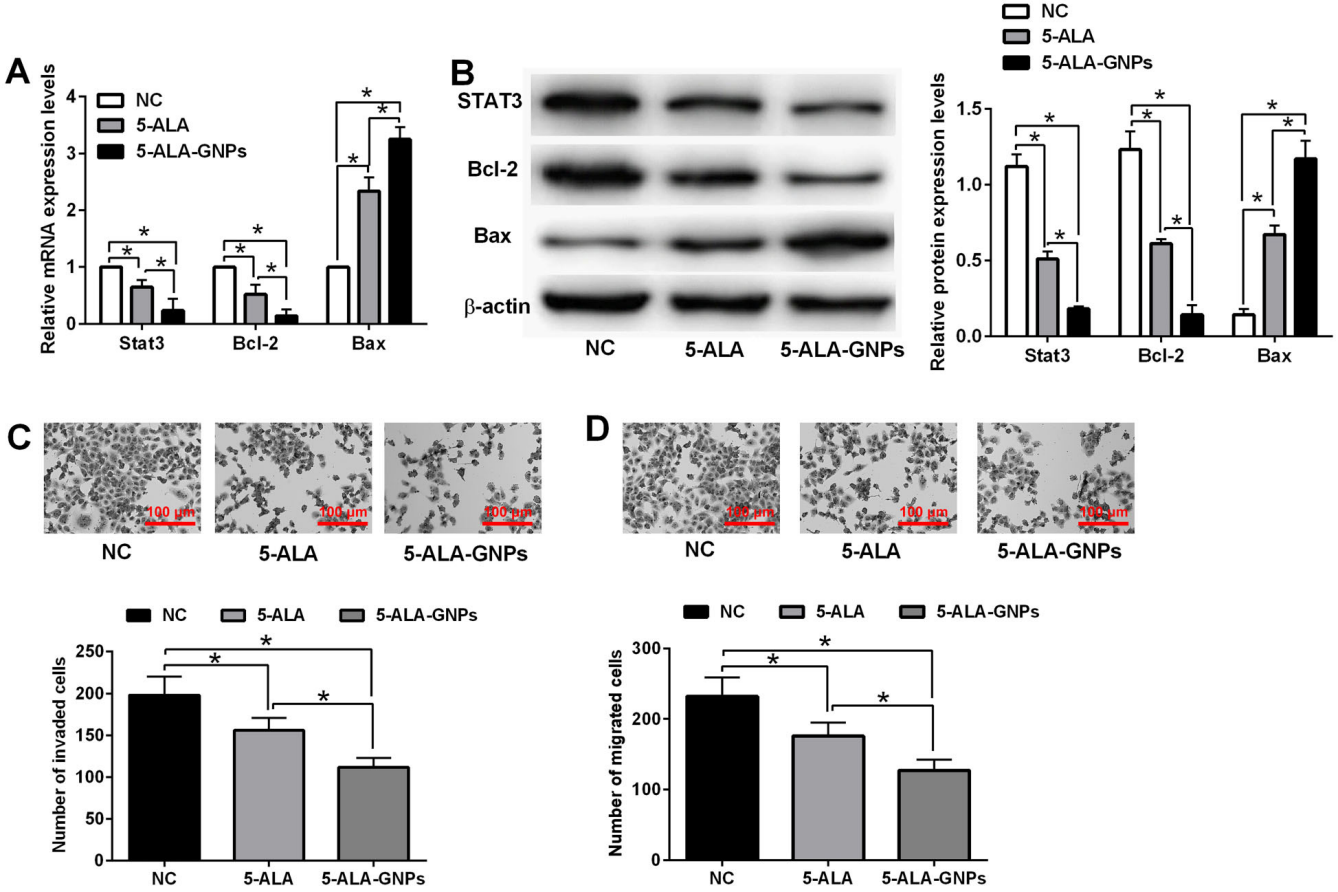
Effects of *in vitro* photodynamic therapy (PDT) on cell invasion and migration in A431 cells. **A**, The mRNA expression and (**B**) protein expression of STAT3, Bcl-2, and Bax in A431 cells after *in vitro* PDT with 5-aminolevulinic acid (5-ALA) (4 mM), 5-ALA-gold nanoparticles (GNPs) (8 mM), or phosphate-buffered saline (PBS (NC)) was determined by qRT-PCR assay (one-way ANOVA). **C** and **D**, Cell invasion and migration of A431 cells after the same treatment were determined by transwell invasion and migration assays, respectively. Scale bars, 100 µm. Data are reported as means±SD (n=3). Significant differences between groups are indicated as *P<0.05 (one-way ANOVA).

The effects of PDT on mRNA and protein expression of STAT3, Bcl-2, and Bax in A431 cells were determined by qRT-PCR and western blot assays, and PDT treatment with 5-ALA and 5-ALA-GNPs significantly suppressed the mRNA and protein expression of STAT3 and Bcl-2 and increased the mRNA and protein expression of Bax in A431 cells. In the 5-ALA-GNPs group, the mRNA and protein expression of STAT3 and Bcl-2 were significantly lower and the mRNA and protein expression of Bax were significantly higher than those in the 5-ALA group (Figure 5A and B).

The effects of PDT on cell invasion and migration of A431 cells were determined by transwell invasion and migration assays. PDT with 5-ALA and 5-ALA-GNPs both inhibited cell invasion and migration of A431 cells. In addition, the invasive and migratory potentials of A431 in the 5-ALA-GNPs group was much lower than those in 5-ALA group (Figure 5C and D).

### Effects of *in vitro* PDT on Wnt/β-catenin signaling activities in A431 cells

**Figure 6:**
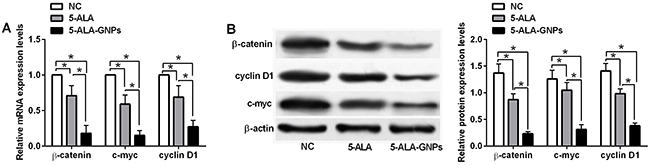
Effects of *in vitro* photodynamic therapy (PDT) on Wnt/β-catenin signaling activities in A431 cells. **A**, mRNA expression and (**B**) protein expression of β-catenin, c-myc, and cyclin D1 in A431 cells after *in vitro* PDT with 5-aminolevulinic acid (5-ALA) (4 mM), 5-ALA-gold nanoparticles (GNPs) (8 mM), or phosphate-buffered saline PBS (NC) was determined by qRT-PCR assay and western blot assay. Data are reported as means±SD (n=3). Significant differences between groups are indicated as *P<0.05 (one-way ANOVA).

The effects of PDT on the Wnt/β-catenin signaling activities in A431 were evaluated by qRT-PCR and western blot assays. PDT with 5-ALA and 5-ALA-GNPs suppressed the mRNA and protein expression levels of β-catenin, c-myc, and cyclin D1 compared to the NC group in A431 cells. Furthermore, the suppressive effects on the expression of Wnt/β-catenin signaling-related markers was potentiated in the 5-ALA-GNPs group compared to the 5-ALA group in A431 cells (Figure 6).

## Discussion

PDT uses light to activate a non-toxic PS to generate reactive singlet oxygen, which mediates toxic effects in the target tumor cells. In clinical practice and *in vitro*, a number of parameters, including the concentration of the PS ALA, the ALA incubation time, and the dose of light are key factors that determine the cytotoxicity of ALA-mediated PDT in tumor cells. Compared to other PSs, 5-ALA offers a number of advantages, such as low toxicity towards normal cells, its short clearance time, and the fact that it is rapidly converted to porphyrin (23). However, the hydrophilicity of 5-ALA and the low permeability of cell membranes to 5-ALA may limit the clinical efficiency of 5-ALA PDT (24). Furthermore, 5-ALA has poor stability under reductive biological conditions (25). Under alkaline conditions, 5-ALA loses hydrogen ions and becomes negatively charged. Thus, these polar characteristics of 5-ALA limit its ability to cross cell membranes. However, conjugation to biocompatible GNPs has been demonstrated to effectively deliver 5-ALA into target cells *in vitro* (23). The GNPs are reduced and become positively charged and are coated with BPEI, which makes 5-ALA-GNPs relatively stable. In our study, no significant ALA was released from ALA-GNPs and the ALA-GNPs were kept stable at 4°C storage. Cheng et al. found that non-covalent drug/GNP conjugates could penetrate into tumors and lead to rapid drug release within hours (26). Zhang et al. (20) found that anti-cancer drugs conjugated to GNPs showed enhanced drug solubility and efficacy by increasing internalization of the conjugates. A recent study showed that 5-ALA conjugated to GNPs showed an enhanced uptake by the cancer cells compared to free 5-ALA (27). These results may imply that the increased tumor suppressive activities of the conjugates may be due to the increased uptake of 5-ALA by cancer cells.

Daniel et al. (28) demonstrated that delivery of a PS using a drug carrier significantly enhanced the inhibitory effects on tumor cell proliferation compared to the PS alone. In the present study, 5-ALA conjugated to GNPs improved the membrane permeability of 5-ALA, and 5-ALA-GNPs could stably accumulate in A431 cells and induce a dual photodynamic and photothermal effects, which could potentiate the anti-tumor effects of PDT in A431 cells.

PS selectivity is the most critical issue in PDT sensitizer design and efficiency. The higher the PS selectivity, the greater the accumulation of the PS in cancer cells, which reduces the side effects in normal cells (29). Therefore, HaCat cells were used to assess the effects of 5-ALA-GNP-mediated PDT in normal cells. PDT using 5-ALA-GNPs led to fewer morphological changes, a significantly lower inhibitory rate, and significantly less apoptosis in HaCat cells than A431 cells. Moreover, the levels of singlet oxygen were significantly higher in A431 cells than HaCat cells, indicating that the 5-ALA-GNPs exerted selective effects in tumor cells.

PDT based on 5-ALA induces tumor cell death by both apoptotic and necrotic pathways (30). We showed that 5-ALA-GNPs effectively induced apoptosis in A431 cells in a concentration-dependent manner. Saczko et al. found that induction of apoptosis by PDT in a human melanoma cell line was dependent on photodynamic sensitivity, and that the DNA damages were determined by the PS concentration and duration of irradiation (31). We found that 5-ALA-GNPs induced significantly higher levels of apoptosis in A431 cells than HaCat cells, confirming the selective effects of 5-ALA-GNPs in tumor cells compared to normal cells. These findings imply that 5-ALA-GNPs could be used to selectively target tumor cells and have limited effects on normal cells.

PDT has been proposed to induce apoptosis in tumor cells by two mechanisms: 1) endogenous mechanisms, mediated by the loss of mitochondrial membrane integrity and increased mitochondrial membrane permeability; and 2) exogenous mechanisms, mediated by the endoplasmic reticulum stress-induced pathway (32). Zhang et al. (27) reported that the enhanced tumor cell-killing efficiency of 5-ALA-GNP conjugate-based PDT was mainly due to improved delivery of the PS to cells by the GNPs. In this study, 5-ALA-GNPs led to significantly higher singlet oxygen generation than 5-ALA. The conversion of 5-ALA into PpIX and generation of singlet oxygen mainly occur in the mitochondria, which is critical to improve efficiency and minimize unwanted cytotoxic activity of PDT (33). In this regard, improving mitochondria localization of 5-ALA is important. The enhanced membrane permeability of 5-ALA-GNPs plays a critical role in increasing the delivery of PS into the mitochondria. 5-ALA-GNPs accumulate more efficiently in the mitochondria, at least in part, as positively charged and cationic GNPs are delocalized and have the potential to cross the inner mitochondrial membrane (34).

One possible mechanism of action of PDT is related to altered expression of Bcl-2 and Bax, which results in the inhibition of the JAK-STAT pathway (35). However, the specific mechanism by which 5-ALA-GNPs enhance PDT has not been fully elucidated. In this study, the flow cytometry results showed that cells treated with 5-ALA-GNPs after PDT had increased cell apoptotic rates compared to cells treated with 5-ALA after PDT, and the effect was enhanced with the increasing concentrations of 5-ALA. In addition, qRT-PCR and western blot were employed to explore the effects of 5-ALA-GNP-induced PDT in A431 cells. Compared to cells treated with 5-ALA, cells treated with 5-ALA-GNPs had significantly lower expression levels of STAT3 and Bcl-2 and higher expression levels of Bax, suggesting the induction of the apoptosis pathway and inhibition of the JAK-STAT pathway. In addition, 5-ALA-GNPs treatment enhanced the inhibitory effects of PDT on cell invasion and migration and Wnt/β-catenin signaling activities in A431 cells compared to 5-ALA treatment. Jiang et al. (36), showed that PDT with hypocrellin B inhibited cell migration in ovarian cancer. PDT also possesses inhibitor effects on cell invasion and migration of head and neck cancer cells *in vitro* (37). PDT with 5-ALA also induced phenotypic change and suppressed migration in human tongue squamous carcinoma cells (38). Wnt/β-catenin signaling has been suggested for its important role in the cancer metastasis. Studies by Ma et al. (39) showed that nanoparticle delivery of Wnt-1 siRNA enhanced PDT by inhibiting the epithelial-mesenchymal transmission for oral cancer. In addition, Dickkopf 3, a Wnt signaling inhibitor, intensified the anti-tumor effects of PDT on breast cancer cells (40). Collectively, these data may imply that 5-ALA-GNPs potentiate the inhibitory effects of PDT on A431 cell invasion and migration possibly via Wnt/β-catenin signaling.

The present study demonstrated that 5-ALA conjugated to GNPs significantly enhanced the efficacy of 5-ALA PDT in A431 cells. Furthermore, this study provided basic optimal parameters and a better understanding of the mechanism of action of 5-ALA-GNP-induced PDT in A431 cells. Further *in vitro* and clinical studies are required to determine the safety and efficacy of 5-ALA-GNPs as treatment for cutaneous squamous cell carcinoma. Indeed, novel PS functions in the tumor microenvironment need to be designed to improve the clinical efficacy of PDT and the outcomes of patients with cutaneous squamous cell carcinoma.

## Supplementary Material

Click here to view [pdf].
